# Amateurism in flux: NIL beyond borders and the strategic crossroads of European basketball

**DOI:** 10.3389/fspor.2025.1690859

**Published:** 2025-11-05

**Authors:** Dimitrios I. Bourdas, Panteleimon Bakirtzoglou, Apostolos Theos, Antonios K. Travlos

**Affiliations:** ^1^ Section of Sport Medicine & Biology of Exercise, School of Physical Education and Sports Science, National and Kapodistrian University of Athens, Daphni, Greece; ^2^ School of Physical Education and Sport Science, Aristotle University of Thessaloniki, Thessaloniki, Greece; ^3^ Section of Sports Medicine, Department of Community Medicine and Rehabilitation, Umeå University, Umeå, Sweden; ^4^ Umeå School of Sport Sciences, Umeå University, Umeå, Sweden; ^5^ Department of Sports Organization and Management, Faculty of Human Movement and Quality of Life Sciences, University of Peloponnese, Sparta, Greece

**Keywords:** talent transition, talent development, talent identification, basketball recruiting, sports marketing, sports product, NCAA, profiling—monitoring

## Key points

This article examines how the NCAA's Name, Image, and Likeness (NIL) policy is reshaping the global basketball landscape by offering European athletes new incentives to pursue U.S. college pathways, blending sport, education, and early brand monetisation. It highlights how this shift challenges the traditional European club-based development model, potentially weakening domestic talent pipelines while opening space for new forms of athlete support and transatlantic cooperation. The study argues that NIL should not be viewed merely as a commercial reform but as a broader cultural and structural inflection point—requiring proactive, context-specific strategies to safeguard the sustainability of European basketball.

## From sacred olive wreaths to sponsored Jerseys: a historical introduction to collegiate basketball athlete compensation and prestige

The NCAA's adoption of its Name, Image, and Likeness (NIL) Interim Policy in July 2021 marked a paradigm shift in collegiate sport (1). Driven by legal reinterpretations of amateurism and athlete rights, the policy allows student-athletes to monetise their personal brands without forfeiting eligibility (2). While this reform originated in the American collegiate context, its ripple effects are increasingly evident across the global sports landscape (3–5)—most notably in basketball (6, 7).

Basketball's international reach (8), coupled with its dynamic interplay of technical, tactical, and developmental demands (9–15), makes it especially susceptible to shifts in athlete mobility and compensation. The NIL era introduces new economic incentives during athletes' formative years, reshaping longstanding developmental models—particularly in Europe, where a historically club-based system has nurtured elite talent through academy structures and regulated pathways to the professional and NBA levels (16).

However, the linkage between athletic excellence, public prestige, and economic reward is hardly novel (17). In antiquity, victors at the Panhellenic Games, including Olympia, often received substantial material rewards in addition to symbolic crowns—such as pensions, tax exemptions, and even statues or poetic tributes. These practices illustrate an enduring logic: athletic glory has long been convertible into political, economic, and cultural capital.

NIL policies represent a contemporary articulation of this same logic. But in contrast to the state-backed sponsorship of ancient athletes, NIL privileges individual marketability—embedding commercial considerations within the structure of athlete development itself. This poses new challenges to European basketball, where institutions must now compete not just on athletic merit but also on economic and symbolic value propositions. NCAA programs, with their global media reach and capacity for brand monetisation, increasingly appeal to elite European prospects and their families, altering the cost-benefit calculus of remaining in Europe (3, 6, 18–21).

This shift threatens to undermine key pillars of European basketball: the economic viability of youth development pipelines, the competitiveness of domestic leagues, and the alignment between athlete aspirations and institutional offerings. Yet despite growing media attention and policy discourse surrounding NIL (22) and the clear relevance of NIL to global talent flows, academic research has largely remained U.S.-centric (23–25), with little attention to how these dynamics are reconfiguring development, governance, and mobility outside North America.

**Figure 1:**
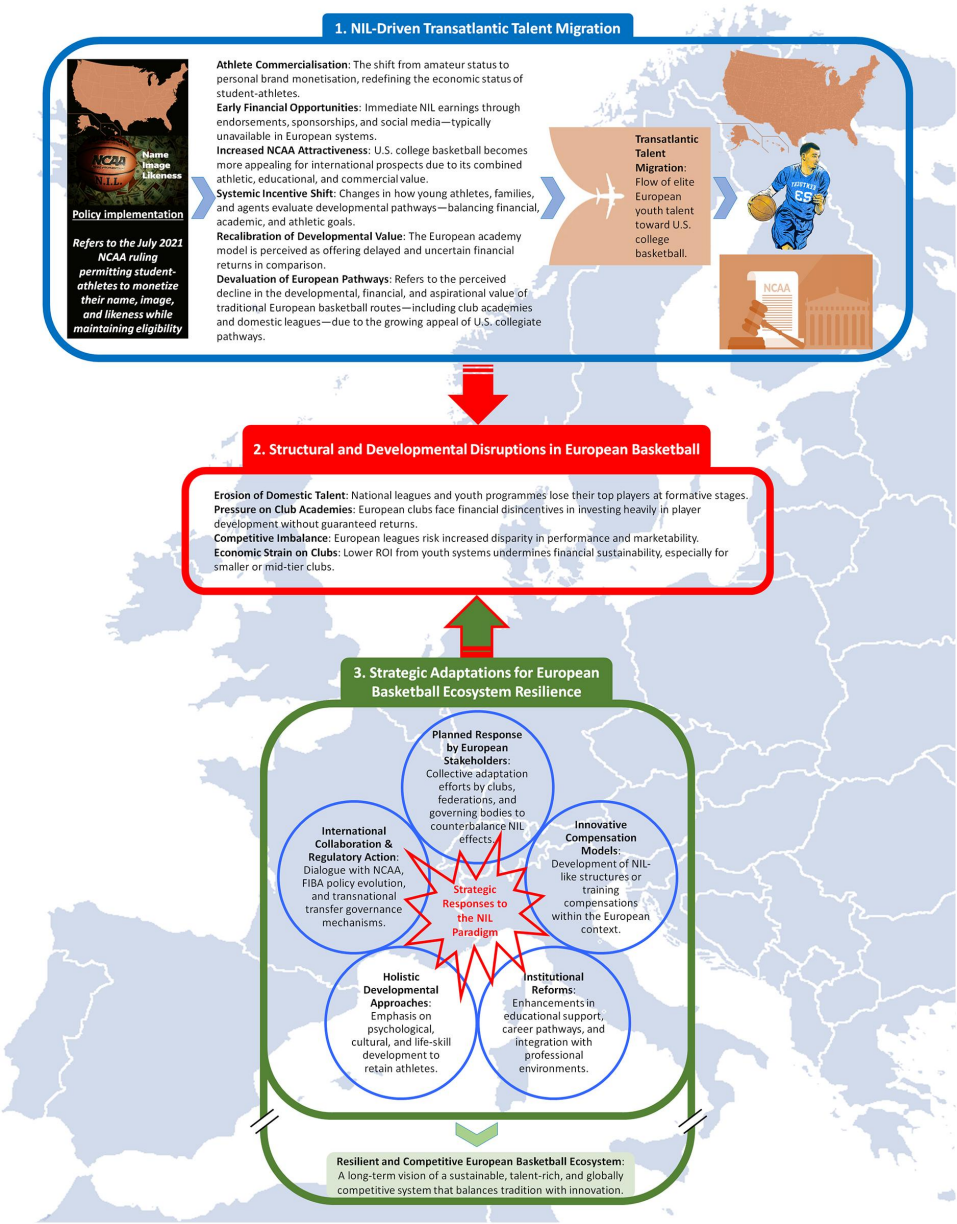
Conceptual illustration of the transatlantic impact and strategic response to the NCAA NIL policy in European basketball. 1. Observed athlete movement patterns from Europe to U.S. collegiate basketball in response to NIL-related incentives; 2. Potential systemic impacts on talent pipelines, club economics, and institutional stability across the European basketball ecosystem; 3. Proposed stakeholder responses aimed at sustaining competitiveness and mitigating the effects of NIL-induced asymmetries. FIBA, international basketball federation; NCAA, national collegiate athletic association; NIL, name, image, and likeness; ROI, return on investment.

This article seeks to address that gap. It does not aim to deliver definitive empirical conclusions, given the recency of NIL and the limited longitudinal data. Rather, it offers a conceptual and critical exploration of NIL's implications for European basketball—identifying emergent trends, institutional pressures, and future research trajectories. By theorising NIL as both a disruptive and generative force, this study provides a novel analytical lens through which to examine how evolving athlete compensation models are reshaping the global basketball ecosystem (Figure 1).

## Transatlantic divergence: structural and cultural contrasts between NCAA and European basketball systems

Understanding the implications of NIL for European basketball requires not only conceptual framing but also attention to the structural and legal underpinnings of each system. In particular, the contrasts between the NCAA's centralised, education-driven model and Europe's decentralised, market-orientated framework highlight how NIL reconfigures existing asymmetries in athlete development and mobility.

The NCAA operates within a highly centralised architecture, governed by uniform eligibility rules, academic integration mandates, and an ethos of amateurism that has been consistently codified through regulatory enforcement and case law (1, 2, 26, 27). By contrast, European basketball follows a decentralised, club-based model shaped by national labour laws and EU employment directives, where athletes often sign remunerated contracts in adolescence (28–30). This divergence reflects not merely organisational form but fundamentally different institutional logics: the NCAA frames athletes as students with conditional access to monetisation, whereas European clubs frame them as workers embedded in professional markets (31).

Strategically, the introduction of NIL does not so much commercialise European basketball—which has long operated under market principles—but rather generates new forms of competitive asymmetry. Recent NCAA recruitment cycles illustrate this imbalance: since the introduction of NIL in 2021, the number of European players on NCAA Division I rosters has risen to over 600, with top-tier prospects increasingly citing NIL opportunities as a decisive factor in their migration (32). This outflow alters the balance of incentives, as U.S. programs can now combine education, global visibility, and immediate commercial rewards in ways European clubs struggle to replicate.

## Financial, academic, and athletic utility: a triadic model of NIL's potential influence

The NCAA's interim NIL policy marks a structural inflection point for elite European basketball prospects, inserting a third pathway into the traditional binary of early professionalisation in Europe vs. U.S. collegiate amateurism (19, 21). By allowing student-athletes to monetise their personal brand while retaining NCAA eligibility, NIL introduces a new calculus that can be conceptualised through three interlinked utilities: financial, academic, and athletic (1, 2). This triadic lens aligns with human capital theory (33, 34), which emphasises how investments in education, skill acquisition, and market visibility yield both immediate and long-term returns. It also resonates with established analyses in sports economics that view athlete migration as shaped by opportunity structures balancing financial incentives, developmental environments, and career security (28, 35, 36).

Financially, NIL disrupts the long-standing dominance of European clubs over early-career compensation. Through endorsements, social media, and sponsorships, collegiate athletes—especially those with transnational appeal—can generate income that rivals or surpasses entry-level contracts in Europe (1, 2). Several recent cases illustrate this trend: Aday Mara reportedly commanded a $700,000 buyout clause to join UCLA (37); Kasparas Jakucionis declined a potential senior role at Barcelona in favour of a reported six-figure NIL package at Illinois; and Egor Demin, previously with Real Madrid, opted for BYU amid substantial NIL opportunities. While these are individual cases rather than systematic data, they highlight a broader shift in valuation and migration dynamics. NIL also reduces the economic risk of delaying professionalisation (19), making NCAA enrolment more appealing for players and potentially influencing agent behaviour—thereby challenging club authority over talent pipelines.

Academically, the U.S. system offers a more robust and institutionally supported dual-career model than most European pathways (38). Access to internationally recognised degrees, academic support structures, and broader cultural immersion enhances long-term career security and holistic development—key considerations for players and families weighing uncertain athletic outcomes (18, 34).

Athletically, the NCAA remains a powerful gateway to the NBA, offering unmatched visibility through televised competitions, social media amplification, and signature events like March Madness (39). For many scouts and agents, NCAA basketball better approximates the physicality and pace of the NBA than European youth or secondary leagues. Coupled with superior facilities and sports science infrastructure, this positions NCAA programmes as not just an alternative—but a developmental upgrade for some.

While the relative value of these three mechanisms varies by individual context, their convergence under NIL has reshaped the strategic landscape of European basketball development—altering incentives for players, agents, and institutions alike.

### Economic and developmental strain in the wake of NIL policy implementation

The rise of NIL as a legitimate income stream for European basketball prospects represents a structural shock to the economic and developmental logic underpinning Europe's club-based talent systems (3, 7, 19, 40). Historically, elite academies have invested heavily in early-stage development—justified by long-term returns through integration into senior squads or lucrative buyout fees. With NIL now offering competitive compensation via endorsements and sponsorships, that rationale is weakening (41).

Recent high-profile cases—such as Aday Mara (UCLA), Kasparas Jakucionis (Illinois), Egor Demin (BYU), and Tomislav Ivišić (Illinois)—as well as emerging commitments from prospects like Dame Sarr (Duke), Vangelis Zougris (Louisville), Luka Bogavac (North Carolina), and others, illustrate a growing tendency for elite European talent to choose NCAA pathways. While these examples are anecdotal rather than systematic, they highlight an early directional shift: the NCAA's ability to combine immediate financial benefits with educational and athletic progression is altering decision-making at the upper tiers of youth development. Early roster data support this trend: since the implementation of NIL in 2021, the number of European players on NCAA Division I men's basketball rosters has grown from approximately 500 to over 600 by the 2024–25 season, marking a 20% increase (32).

This trend has raised concerns among European stakeholders about reduced bargaining power, weakened buyout enforcement, and pressures on youth salary structures. Some reports suggest clubs have begun offering higher stipends or early professional contracts to retain talent (42, 43), though comprehensive data documenting this adjustment remain limited. The possibility of “defensive escalation” in youth salaries therefore requires careful longitudinal study to assess its scale and sustainability.

Beyond economics, developmental continuity is also at risk. European academies emphasise long-term tactical and physical progression, but early departures truncate this arc, leaving clubs without return and NCAA programmes responsible for incomplete development. Domestic leagues, in turn, risk diminished competitive depth, while coaches and younger players confront eroded trust in the system. Reintegration challenges for players returning from U.S. programmes—owing to differing tactical styles, income expectations, and professional norms—further complicate the developmental equation (44).

In response, early proposals have surfaced: stronger pre-professional contracts, pan-European coordination, and FIFA-style training compensation mechanisms (42, 43). Yet absent systemic reform, the current model faces a growing risk of fragmentation—economically unsustainable, developmentally porous, and culturally destabilised.

### NIL and the transformation of amateurism: stakeholder perspectives in European basketball

The implementation of NIL rights in U.S. collegiate athletics presents both novel opportunities and significant challenges for European basketball. To analyse these effects, we adopt a stakeholder theory perspective (45–47), which emphasises the interdependent interests of multiple actor groups in shaping institutional change. Building on the stakeholder salience framework (48), we identify three highly salient stakeholders—athletes, clubs, and the broader European basketball ecosystem—whose positions are most directly influenced by NIL policy developments.

**Figure 2:**
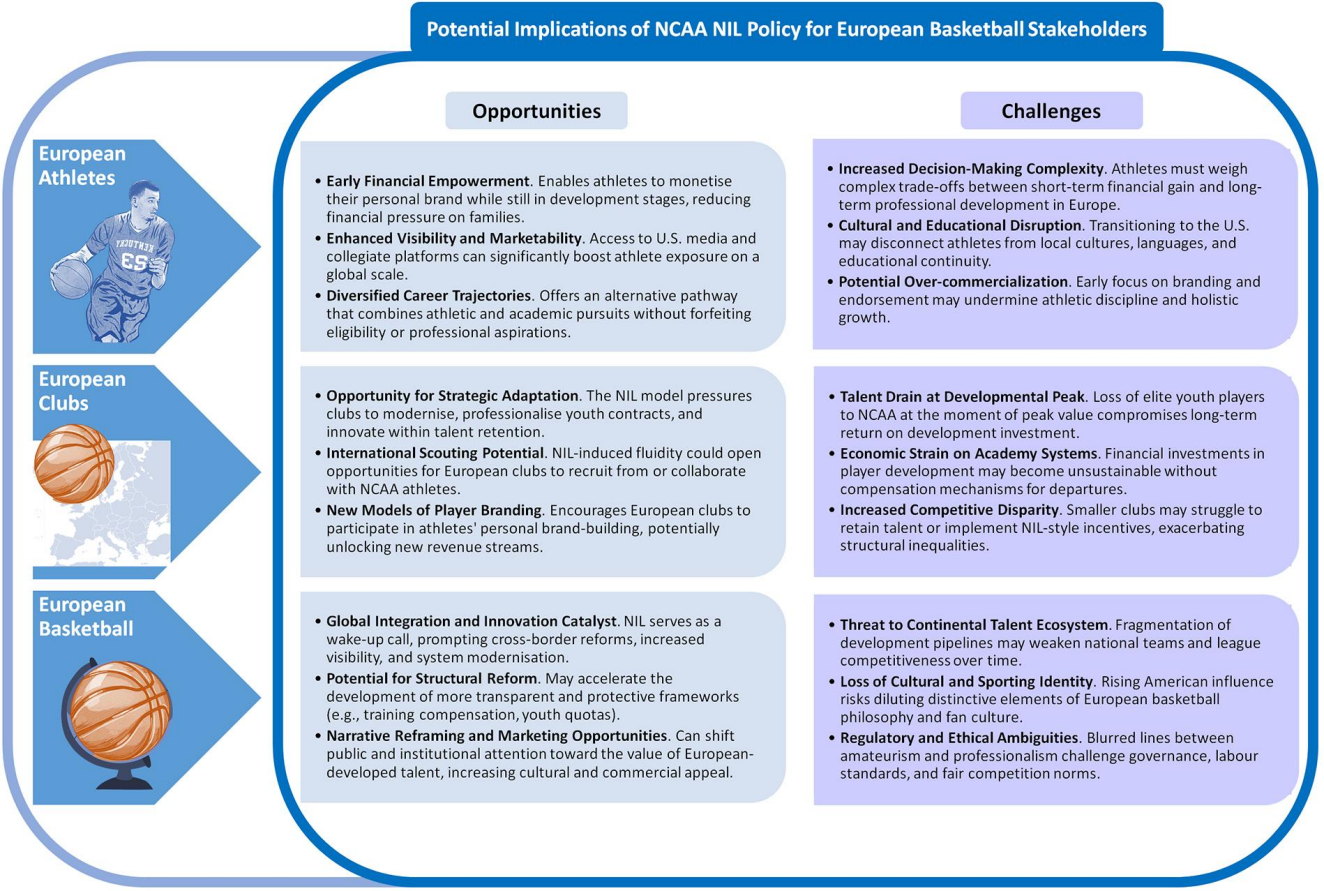
Potential implications of the NCAA NIL policy for three key European basketball stakeholder groups—athletes, clubs, and the sport as a whole—structured using principles from stakeholder theory (45, 48). The diagram synthesises economic, developmental, and structural dimensions to illustrate how the NIL paradigm may influence transatlantic talent mobility, institutional strategy, and the evolving concept of amateurism within the European basketball ecosystem. NCAA, national collegiate athletic association; NIL, name, image, and likeness.

The accompanying infographic (Figure 2) synthesises the core benefits and risks associated with NIL across these three groups, highlighting its economic, developmental, and structural implications. This systematisation situates NIL not merely as an individual opportunity but as a transnational force reshaping incentives, institutional strategies, and the evolving conception of amateurism in European sport.

### For European athletes

The most immediate benefit of NIL for European athletes is the economic agency it grants (24). For decades, student-athletes contributed to revenue-generating systems without compensation commensurate with their labour. NIL disrupts this status quo by allowing athletes to monetise their personal brand through endorsements, sponsorships, and digital media while retaining NCAA eligibility (49). This income stream provides financial stability, particularly for those from modest socio-economic backgrounds, and may rival or exceed early-career earnings in European professional leagues.

Beyond financial gains, NIL participation cultivates skills in financial literacy, negotiation, entrepreneurship, and brand management—competencies transferable to both professional sport and post-athletic careers. Academically, the NCAA offers accredited degrees and dual-career support (24, 50), safeguarding against uncertain athletic outcomes. Athletically, NCAA participation enhances visibility through media exposure, especially during major tournaments (39), and NIL further amplifies this reach via social media channels, increasing sponsor appeal and market value (49).

However, NIL also introduces risks. Financially incentivised performance expectations can generate psychological strain (51–56), anxiety, and burnout (5, 55). These pressures are compounded by cultural dislocation, social media scrutiny, and time management challenges, particularly for international athletes adjusting to the American collegiate model (57, 58). Earnings disparities between high- and low-profile athletes may also undermine team cohesion (40, 54), while short-term NIL earnings could distort long-term career decisions. For European athletes unfamiliar with such dynamics, this can hinder integration and development (59). Reintegration into European systems post-college can also be difficult due to differences in playing style, tactical expectations, and salary structures (44). Legal ambiguities further complicate matters. International athletes on F-1 visas face potential conflicts between NIL income and immigration restrictions, threatening both eligibility and legal status (5, 53, 54, 60). Additionally, disparities in NIL resource distribution have fostered quasi-professional dynamics within NCAA recruiting, undermining the principle of competitive equity (7, 23).

### For European basketball clubs

For clubs, NIL introduces both risks and opportunities. On the positive side, NCAA participation offers an extended evaluation window, allowing clubs to monitor European athletes in competitive, well-resourced environments before committing to professional contracts (6, 21). Players returning from NCAA programmes may bring enhanced physical conditioning, media skills, and commercial awareness (44). NIL success can also serve as a proxy for off-court professionalism. Moreover, the NIL era could catalyse transatlantic cooperation. Strategic partnerships between NCAA programmes and European clubs may support talent pipelines, post-college placement schemes, and shared scouting systems. In parallel, NIL pressures may drive European clubs to invest in academic integration, life-skills programming, and holistic development to improve retention.

Nonetheless, the structural threats are considerable. European clubs typically invest in academy talent with the expectation of either first-team integration or transfer compensation. NIL incentivises early departures to the NCAA, often without financial return. This undermines the sustainability of current development models and may prompt clubs to downscale or restructure academies.

The appeal of NCAA pathways—driven by higher short-term earning potential—can erode domestic talent pools and disrupt roster planning. In response, clubs may lean on short-term signings or premature promotions, jeopardising competitive stability. Additionally, stylistic convergence with NCAA norms may dilute the technical-tactical identity of European basketball.

Furthermore, the absence of a transatlantic compensation mechanism akin to FIFA's training compensation compounds these challenges. Legal protections for youth contracts often do not extend across jurisdictions, making enforcement difficult. Agents, empowered by NIL structures, are incentivised to redirect talent flows towards monetisation rather than long-term development, exacerbating misalignment between athlete goals and club interests.

### For European basketball as a whole

At the ecosystem level, NIL enhances global scouting efficiency, making European players visible in a data-rich U.S. environment. The success of NIL-enabled athletes can elevate the prestige of European basketball, attract corporate sponsorship, and foster intercontinental collaboration. Athletes returning from NCAA programmes often bring entrepreneurial skills in branding and media management that enrich the European sport economy.

Yet structural downsides are significant. NIL intensifies transatlantic migration, weakening domestic pipelines and reducing league competitiveness (61). European clubs receive little return on grassroots investments, threatening the long-term viability of development structures. The growing emphasis on individual brand monetisation risks undermining collective, team-orientated values historically embedded in European sport. Finally, the absence of a coordinated European response leaves the continent vulnerable to becoming a passive supplier of talent to a commercially stronger U.S. model. Without systemic adaptation, Europe risks institutional fragmentation and cultural destabilisation.

### NIL as systemic disruption: strategic adaptation in European basketball

The rise of NIL in U.S. collegiate sport presents a systemic challenge to European basketball, particularly in terms of talent retention, developmental continuity, and institutional sustainability (49). To remain viable, European stakeholders—including clubs, national federations, and governing bodies like FIBA and EuroLeague—must shift from reactive fragmentation to coordinated structural reform (Figure 1).

A priority is recalibrating the European pathway to retain elite youth athletes. Financial competitiveness is key. Introducing pre-professional contracts with performance incentives and clearer senior integration routes could offer viable alternatives to NCAA migration. These models should be supported by an enforceable training compensation system—similar to FIFA's in football—to secure a return on developmental investment when athletes transfer abroad. FIBA's ongoing work with the NCAA on the “Letter of Clearance” framework may serve as a useful mechanism in this regard (43).

Simultaneously, the European system must strengthen its academic dimension. Partnerships with secondary and tertiary institutions, supported by academic advisors, digital learning platforms, and dual-career pathways, could replicate the NCAA's educational appeal without geographic displacement. Holistic development—through life-skills training, mental health support, and career planning—should be institutionalised to match evolving athlete needs.

On the athletic front, enhancing intra-European mobility and competition visibility is critical. Expanding elite youth tournaments and reforming domestic loan systems can provide high-level exposure while maintaining developmental continuity. Narrative reinforcement also matters: spotlighting homegrown stars like Dončić, Jokić, and Antetokounmpo can counteract perceptions of the NCAA as the singular launchpad to global success.

At the governance level, multi-actor coordination is essential. Aligning youth contract standards, regulating agent behaviour, and enforcing homegrown player quotas could create a more coherent talent ecosystem (62). Moreover, introducing incentives for clubs that successfully graduate academy players into senior roles may reinforce institutional commitment to development (62).

Finally, brand positioning must evolve (63). The European system offers early professionalisation, competitive diversity, and cultural plurality—features that can be strategically packaged and marketed. Facilitating domestic sponsorship opportunities for young talent and investing in league-wide storytelling initiatives may partially replicate NIL's commercial appeal within Europe's existing framework.

### From impact to insight: toward a research agenda on NIL and European basketball

The transformative impact of NIL policy on European basketball demands a systematic research agenda that captures both immediate disruptions and longer-term structural shifts. In the absence of robust longitudinal data, a forward-looking, interdisciplinary approach is critical to guide evidence-based policy and strategic reform. While elements of such an agenda build on existing strands of literature in sport labour migration, dual-career pathways, and athlete welfare, the NIL phenomenon introduces qualitatively new conditions that warrant targeted investigation.

A central priority is quantifying transnational talent migration (20, 44, 64, 65). Tracking player movement from European academies to NCAA programs post-NIL and assessing its effects on player development, club competitiveness, and national team pipelines will establish essential baselines. Complementary qualitative research should explore the motivations and decision-making logics of European athletes, clarifying how academic, athletic, and cultural perceptions of the NCAA influence migratory choices.

Given NIL's financial implications, targeted economic analyses are needed (40, 58, 59, 61, 66). This includes modelling alternative compensation strategies within European systems—such as endorsement-based incentives or training compensation frameworks—and evaluating their feasibility under EU labour law.

Institutional responses also warrant scrutiny (67–69). Case studies of clubs that have retained talent, formed academic partnerships, or innovated contract structures can yield scalable strategies. Similarly, comparative legal research on amateurism, player rights, and governance across jurisdictions can illuminate emerging regulatory tensions and inform transnational coordination.

Beyond economics and law, the sociocultural and ethical dimensions of NIL-induced change must be addressed (35, 36, 51, 70–73). This includes examining its impact on club identity, fan engagement, and equity of access—especially for athletes from under-represented or economically disadvantaged backgrounds.

Finally, the evolving role of agents, shifts in talent valuation, and new career logics merit close examination (25, 38, 44, 49, 64, 65, 69, 74–76). Surveys of players, families, and intermediaries could reveal how the NIL era is reshaping early-career strategy and the perceived value of different developmental routes.

In sum, the proposed research directions are not intended as an entirely new departure, but as a focused extension of existing literature into the NIL era. Together, these research vectors can underpin a more nuanced, empirically grounded understanding of NIL's impact on European basketball—supporting sustainable reform and safeguarding athlete welfare, institutional identity, and competitive balance in an increasingly globalised basketball ecosystem.

## Perspective: navigating the NIL era in European basketball

The NCAA's NIL policy represents a transformative development in global sport, reshaping athlete mobility and value creation beyond the U.S. context. While rooted in American legal and cultural frameworks, its ripple effects are destabilising key pillars of European basketball's economic and developmental infrastructure. By monetising personal brands during formative years, NCAA programmes now offer a compelling alternative to Europe's traditional club-centric pathways—shifting the transatlantic talent contest from post-development transition to early-stage disruption.

Taken together, this analysis highlights how NIL reframes three interlocking dimensions of European basketball: (1) the economic sustainability of youth academies and talent pipelines, (2) the developmental continuity of players moving between systems, and (3) the structural asymmetry between the NCAA's centralised governance and Europe's fragmented regulatory landscape. These dynamics suggest that NIL functions not only as a disruptive shock but also as a catalyst for institutional learning and adaptation.

At the same time, several limitations of this study must be acknowledged. The analysis relies primarily on secondary sources and illustrative case evidence; systematic longitudinal data on NIL's impact in Europe remains scarce. Moreover, the heterogeneity of European basketball systems—spanning national federations, clubs, and leagues—limits the extent to which uniform conclusions can be drawn. Future research should adopt comparative or mixed-method approaches, integrating quantitative tracking of player migration, salary trends, and club investment strategies with qualitative insights from athletes, agents, and administrators.

Looking forward, the NIL era compels European basketball to rethink its value propositions—not only to remain competitive with the NCAA but also to preserve cultural coherence and long-term viability. Strategic experimentation—such as reinforced pre-professional contracts, enhanced dual-career models, or cross-border compensation mechanisms—may help align incentives across stakeholders. More broadly, Europe's response will hinge on balancing adaptation with identity: maintaining the distinctive features of its sporting culture while engaging productively with the commercial realities of a globalised basketball economy.
